# The Potential Role of Nrf2 Signaling in Leishmania Infection Outcomes

**DOI:** 10.3389/fcimb.2019.00453

**Published:** 2020-01-10

**Authors:** Aislan de Carvalho Vivarini, Ulisses Gazos Lopes

**Affiliations:** Laboratory of Molecular Parasitology, Center of Health Science, Carlos Chagas Filho Biophysics Institute, Federal University of Rio de Janeiro, Rio de Janeiro, Brazil

**Keywords:** Nrf2 signaling, immune system modulation, protozoa, *Leishmania*, gene expression

## Abstract

Nrf2 [nuclear factor erythroid 2-related factor 2 (Nrf2)] regulates the expression of a plethora of genes involved in the response to oxidative stress due to inflammation, aging, and tissue damage, among other pathological conditions. Deregulation of this cytoprotective system may also interfere with innate and adaptive immune responses. Oxidative burst, one of the main microbicidal mechanisms, could be impaired during initial phagocytosis of parasites, which could lead to the successful establishment of infection and promote susceptibility to diseases. A wide diversity of infections, mainly those caused by intracellular pathogens such as viruses, bacteria, and protozoan parasites, modulate the activation of Nrf2 by interfering with post-translational modifications, interactions between different protein complexes and the immune response. Nrf2 may be induced by pathogens via distinct pathways such as those involving the engagement of Toll-like receptors, the activation of PI3K/Akt, and endoplasmic reticulum stress. Recent studies have revealed the importance of Nrf2 on leishmaniasis. This mini-review discusses relevant findings that reveal the connection between Leishmania-induced modifications of the host pathways and their relevance to the modulation of the Nrf2-dependent antioxidative response to the infection.

## Introduction

Cells are constantly being exposed to different conditions that can cause irreversible damage, such as xenobiotics, drugs, and UV rays, as well as endogenous products, such as reactive oxygen species (ROS) (Rahman, 2007). If cells are not checked constantly, these stresses may eventually lead to diseases or different types of infections caused by a variety of pathogens. To protect themselves from such problems, cells have developed mechanisms that prevent substances from the toxicity induced by stress producers, including several phase II defense genes (Kwak et al., 2001). All these defense genes are regulated by the transcription factor Nrf2 (nuclear factor (erythroid-derived 2)-like 2), which is the main cellular mediator during cell adaptation to oxidative stress (Kaspar et al., 2009). In the Nrf2 signaling pathway, an arsenal of antioxidative responsive genes is either constitutively expressed or induced, including those with regulatory activity of an element in the promoter called ARE (Antioxidant Responsive Element) (Wasserman and Fahl, 1997).

The regulation of Nrf2 canonical signaling is primarily undertaken by its inhibitor Keap1 (Dhakshinamoorthy and Jaiswal, 2001). The Nrf2/Keap1 protein complex is a potent oxidative sensor. Under normal conditions, Keap1, together with ubiquitin E-ligase Cullin-3, continually traffics Nrf2 to the 26S proteasome for degradation. In response to different types of stresses, Nrf2 is dissociated from its inhibitor through distinct post-translational modifications in Keap1 and Nrf2, which leads to its localization in the nucleus where it coordinates the expression of several genes. The Nrf2 polypeptide chain is phosphorylated at multiple sites, mainly by PKC-zeta, PERK, GSK3, and several MAPKs (Bloom and Jaiswal, 2003; Sun et al., 2009). Nrf2 regulates approximately 200 genes directly, and as a result, some studies have analyzed the interactions of Nrf2 on a global scale, identifying interactome networks, and Nrf2-associated regulated functions (Türei et al., 2013).

Nrf2 is closely associated with the immune response to different types of antigens and the convergent signaling during pathogen recognition (Battino et al., 2018; Mohan and Gupta, 2018; Olagnier et al., 2018). By presenting an ambiguous response, that is, a response that favors either advanced infection or induces the microbicidal activity of defense cells, Nrf2 may be the crucial target of future pharmacological interventions (Deramaudt et al., 2013; Shah et al., 2018; Cuadrado et al., 2019). The molecular mechanisms of resistance conferred by Nrf2 in response to different levels of stress, such as that from intracellular infections, may vary according to the pathogen species and its virulence, as well as the host immune response, but there remain crucial processes that have not been very well-established and issues that need further study and analysis. This minireview aims to discuss the ways that the transcription factor Nrf2 is involved in infections by *Leishmania* parasites and the signaling mechanisms of Nrf2 that may dictate the modulation of the immune response.

## Nrf2 and the Immune System Modulation

The interest in the mechanisms that promote oxidative stress, a common condition in several critical pathologies, including immunological diseases, has been accompanied by increasing research in Nrf2 signaling. Several authors have attributed an anti-inflammatory profile to Nrf2, indicating its role in the transcriptional, and post-translational inhibition of some components of the inflammatory system, such as NFκB, proinflammatory cytokines, autophagy factors, and Toll-like receptors (Kobayashi et al., 2016; Kapuy et al., 2018; Rubio et al., 2018). The balance between the antioxidant and pro-inflammatory profile promoted by Nrf2 makes it the center of attention when the immune system is modulating infectious pathologies.

Nrf2 is associated with the Th2-related induction of immune cells. Use of tBHQ, a food preservative, and classic Nrf2 inducer, to treat T-CD4+ cells promoted their differentiation such that they expressed a Th2 profile, producing low levels of IFN-γ, and high levels of IL-4, IL-5, and IL-13 (Rockwell et al., 2012). The interaction between NFκB-p65 and Keap1 suppresses the Nrf2-ARE pathway, which represses various genes (Yu et al., 2011). In addition to NFκB, Keap1 also targets IKK-β kinase for degradation, which prevents the activation of NFκB (Kim et al., 2010; Tian et al., 2012). However, NFκB can act directly on the Nrf2 promoter, regulating the induction of Nrf2 transcription (Hayes and Dinkova-Kostova, 2014), and furthermore, several papers have described the close relationship between NFκB pathway signaling and the modulation of Leishmania infection (Calegari-Silva et al., 2009; Pereira et al., 2010).

The PI3K/Akt signaling pathway has been related to Nrf2 activation in many study models. Inhibitory phosphorylation of GSK3 promotes the activation of Nrf2 by inhibiting phosphorylation-induced signaling, thus allowing it to remain stable and active (Chowdhry et al., 2013). In addition, the PI3K/Akt pathway has also been linked to susceptibility to *Leishmania amazonensis* infection, resulting in host cell resistance to apoptosis, and blocked IL-12 expression (Ruhland et al., 2007; Calegari-Silva et al., 2015).

The autophagic degradation of Keap1 enables continued cellular redox homeostasis. The p62/SQSTM1 protein plays a central role in regulating Keap1/Nrf2 signaling. Oxidative stress is decreased in cells overexpressing p62, which increases protein processing through autophagosomes (Darvekar et al., 2014; Rubio et al., 2014). The engagement of TLR4 by LPS induces the activation of p38-MAPK kinase, which leads to the accumulation of Nrf2 in the nucleus and p62 expression (Fujita and Srinivasula, 2011).

Activation of TLR2 culminates in the polarization of macrophages into the M2 phenotype, which causes lysosomal NF-kB-p65 degradation via p62/SQSTM1 during selective autophagy (Chang et al., 2013). This polarization into the M2 phenotype is characterized by the emergence of a type of macrophages called MOX, which has redox and antioxidant potential and induces the expression of the anti-inflammatory and antiapoptotic Cox2, IL1β, HO-1, VEGF, and Nrf2 (Kadl et al., 2010). In human tracheal smooth muscle cells, treatment with LTA (lipoteichoic acid), a gram-positive bacterial cell wall component and a TLR2 agonist, induces HO-1 gene expression via Nrf2 signaling, which leads to the accumulation of HO-1 (Lee et al., 2008). Other studies also demonstrated the participation of TLR4, TLR7, and TLR9 in the regulation of Nrf2, and HO-1 through a mechanism mediated by Btk kinase (Bruton's tyrosine kinase) (Vijayan et al., 2011). After TLR4 activation in sepsis conditions, Keap1^−/−^ mice were more resistant to infection and had increased macrophage capacity (Kong et al., 2010). The regulation of Nrf2 by the hepatitis C virus can be mediated by the activation of PERK following endoplasmic reticulum stress, leading to Mdm2-mediated retinoblastoma protein degradation and subsequent generation of oncogenic stress in HCV-infected cultures (Aydin et al., 2017).

## Nrf2 Pathway in Leishmania Infection

Although infections can cause oxidative bursts, several derivatives of ROS act as signaling molecules that can lead to eventual cellular homeostasis (D'Autréaux and Toledano, 2007). On the basis that different pathogens are favored or restricted by ROS (Paiva and Bozza, 2014), the pathogens and host cells have developed a coevolutionary pattern of subversion in the production of these radicals. For example, *Trypanosoma cruzi* that infect THP-1 cells require oxidative stress to establish a successful parasitic relationship since overexpressed Nrf2 reduces parasitism (Paiva et al., 2012). Nrf2 participates in infections by protozoan microorganisms in addition to those of *Leishmania* spp., such as those caused by *Entamoeba histolytica, Cryptosporidium parvum, Plasmodium* spp., and *Toxoplasma gondii*, all of which culminate in the modulation of Nrf2, and a diminished anti-inflammatory, and antioxidant profile (Morada et al., 2013; Aldaba-Muruato et al., 2017; Ramos et al., 2019; Xu et al., 2019).

Infection with *L. amazonensis* usually induces very low oxidative stress (Almeida et al., 2012). *L. amazonensis* infection, compared to that of *L. major*, produces reduced levels of reactive oxygen intermediates, such as hydrogen peroxide, which is ~20 times lower than that generated during *L. major* infection. Although there are no data on Nrf2 activation levels during *L. major* infection, the reduced ROS production levels caused by *L. amazonensis* infection may reflect the activation of the Nrf2 pathway and, as a consequence, the reduction of cellular oxidative stress (Vivarini et al., 2017). However, the combined treatment of IFN-gamma with LPS induces a high level of oxidative stress with the production of superoxide and nitric oxide, resulting in the elimination of the parasites (Mukbel et al., 2007).

The replication and initial establishment of *L. amazonensis* infection after IFN-I treatment is partly dependent on the enzyme superoxide dismutase 1 (SOD1), which reduces the oxidative stress that is unfavorable to Leishmania and may promote the development and affect the outcome of leishmaniasis (Khouri et al., 2009, 2010). The levels of SOD1 in patients with cutaneous leishmaniasis caused by *L. amazonensis or L. braziliensis* are increased and may serve as biomarkers of these infections (Khouri et al., 2014). In addition to these data, the authors confirmed a positive and close relationship between the levels of host SOD1 and those of parasite SOD2/4, demonstrating that the interaction between parasite and host leads to the control of the gene expression of both genomes. Previously, changes in zinc, copper, and iron levels in the serum of patients with cutaneous leishmaniasis have been observed and may suggest that cofactors of antioxidative stress enzymes may play a role in the infection (Pourfallah et al., 2009).

The evidence that *L. amazonensis* infection activates dsRNA-induced kinase (PKR), which promotes the proliferation of this parasite in macrophages (Pereira et al., 2010; Vivarini et al., 2011, 2015, 2017; Barreto-de-Souza et al., 2014; Rath et al., 2019), suggests that *L. amazonensis* has evolutionarily managed to exploit this signaling pathway in the host cell for its own benefit. As PKR is a signal transduction protein, the inference that increased production of inflammatory mediators dependent on this kinase is plausible; for example, some cytokines (IL-10 and IL-27) and antioxidative enzymes (SOD1 and HO-1) favor the establishment of infection and provide an intracellular milieu for the progression of leishmaniasis. In mouse peritoneal and human macrophages linages, infection by *L. amazonensis* leads to the increase of SOD1 expression in a PKR/Nrf2-dependent manner (Vivarini et al., 2017). This species also exploits the PI3K/Akt pathway to induce Nrf2 translocation to the nucleus and bind to ARE in the SOD1, p62, and Nrf2 promoters. Furthermore, the levels of intracellular ROS, nitric oxide, and peroxynitrite are decreased in infected macrophages, while in Nrf2-deficient cells, amastigotes replication is reduced compared to the replication in wild-type cells. In addition to those caused by *L. amazonensis*, infections caused by *L. braziliensis* and specific strains of *L. amazonensis* (from localized or diffuse cutaneous leishmaniasis patients) also positively modulate the PKR/Nrf2 axis.

The close relationship between oxidative stress and the autophagic process has been shown by evidence from many authors (Lee et al., 2012). The NO burst production in *L. amazonensis* and *L. major* infections was reduced by autophagy induction without causing changes to arginase activity (Dias et al., 2018). As *L. amazonensis* infection is able to induce autophagy, which is essential for the progression of the infection (Pinheiro et al., 2009), and the participation of the Nrf2, PKR, and PI3K pathways has been reported in this cellular process; that is, there is a close relationship or convergence of these signaling pathways such that, together, they may trigger autophagy and the creation of an antioxidative profile during *L. amazonensis* infection. Moreover, PKR-deficient cells have suppressed autophagic processes because PKR phosphorylates eIF2α, which plays a key role in the regulation of autophagosome development (Tallóczy et al., 2002).

Macrophages infected with *L. amazonensis* display reduced Keap1 levels, enabling Nrf2 to translocate into the nucleus and modulate ARE dependent-gene expression. *In situ* transcriptome analyses with samples from *L. braziliensis*-infected patients corroborates the role of IFN-1/PKR, PI3K, the antioxidant responsive element (ARE), and autophagy signaling pathways in the infection (Vivarini et al., 2017).

Moreover, in ATF4 (activating transcription factor 4)-knockdown macrophages, infection by *L. amazonensis* caused decreased Nrf2 expression, and nuclear translocation, reduced HO-1 expression, and increased nitric oxide production (Dias-Teixeira et al., 2017). The same work demonstrated that phosphorylation of PERK induced by endoplasmic reticulum stress culminated in Nrf2 activation, which led to ATF4 dimerization in the nucleus, and increased Nrf2/ATF4 regulation of ARE in the HO-1 gene promoter, thereby favoring *L. amazonensis* infection. A key enzyme triggered by cellular stress is HO-1 (heme oxygenase-1), which exhibits anti-inflammatory, and antioxidant properties, and has a catalytic function that favors Leishmania infection (Luz et al., 2012). *Lutzomyia longipalpis* Saliva, a type of phlebotomine sandfly, induces macrophages *in situ*, and in human skin at the bite site to express of Nrf2, and the HO-1 target gene, showing the means by which *Leishmania* infections are transmitted and established by sandfly-borne vectors (Luz et al., 2018).

Despite the similarities of the species of genus *Leishmania*, the species present different patterns of virulence and pathologies according to the immunological profile of the host. In a proteomics analysis comparing *L. amazonensis* and *L. major* infection, the canonical signature of the Nrf2 pathway was prominent in *L. amazonensis*-infected macrophages, as indicated by a significant increase in HO-1, and SQSTM1 (p62) expression, demonstrating that *L. major* does not use this pathway to try to subvert host cell defenses (de Menezes et al., 2019). *L. donovani*-infected macrophages also exploit the activation of Nrf2. Aiming to survive in macrophages, these parasites increase the expression of Tollip (Toll-interacting protein), a negative regulator of the activation of the IL-1R/TLR pathway, through the direct binding of Nrf2 to the Tollip promoter (Parmar et al., 2018).

## Nrf2 as a Possible Therapeutic Target in Leishmania Infection

In the search of possible alternatives for leishmaniasis treatment, some authors have tested exogenous compounds or plant-derived extracts that may target Nrf2 or ARE-responsive genes. *Caryocar coriaceum* extracts, a plant found in the Brazilian Cerrado, has a leishmanicidal effect in the amastigotes and promastigotes forms of *L. amazonensis* through the upregulation of Nrf2/HO-1/Ferritin expression, subsequently reducing the labile iron pool in infected macrophages such that the replication rate of the parasite is reduced (Tomiotto-Pellissier et al., 2018). Dehydroabietic acid (DHA), isolated from *Pinus elliottii*, a conifer belonging to the family Pinaceae, also has *in vitro* antileishmanial action by downregulating Nrf2/Ferritin expression, thereby inducing a pro-oxidant effect and increasing the ROS levels in macrophages, which adversely affects the replication of *L. amazonensis* (Gonçalves et al., 2018).

In cells infected with *L. braziliensis*, treatment with flavonoid quercetin, which has important known biological properties an antioxidant and anti-inflammatory molecule in addition to its that antiprotozoal and antiviral characteristics (Mamani-Matsuda et al., 2004), increased the gene expression of Nrf2 and HO-1, leading to the depletion of available iron for successful of *L. braziliensis* infection (Cataneo et al., 2019). These compounds not only modulate Nrf2 in leishmaniasis but also trans-chalcone, another flavonoid molecule, that directly promotes an apoptosis-like process in the promastigote and amastigote forms of *L. amazonensis*, which leads to a decrease in ROS, nitric oxide, TGF-β, and IL-10, followed by an increase in Nrf2/HO-1/Ferritin expression, modulating the intracellular proliferation of these parasites (Miranda-Sapla et al., 2019).

*Leishmania donovani* infection induces the expression of the LdMRP2 transporter located in the parasite flagellar pocket, conferring resistance due to drug efflux (Chowdhury et al., 2014). This resistance of the parasite is concomitant with the expression of the MRP2 (multidrug resistance protein 2) transporter in macrophages with a gene that has an ARE in its promoter, and the infection induces the binding of Nrf2. Miltefosine used in cells derived from spleen aspirate and bone marrow infected with *L. donovani* induced oxidative stress and reduced the levels of HO-1, SOD, GPx, and nuclear Nrf2, which led to suppressed IL-10 and TGF-β levels and upregulated expression of IL-12 and TNF-α (Das et al., 2013).

## Discussion

**Figure 1:**
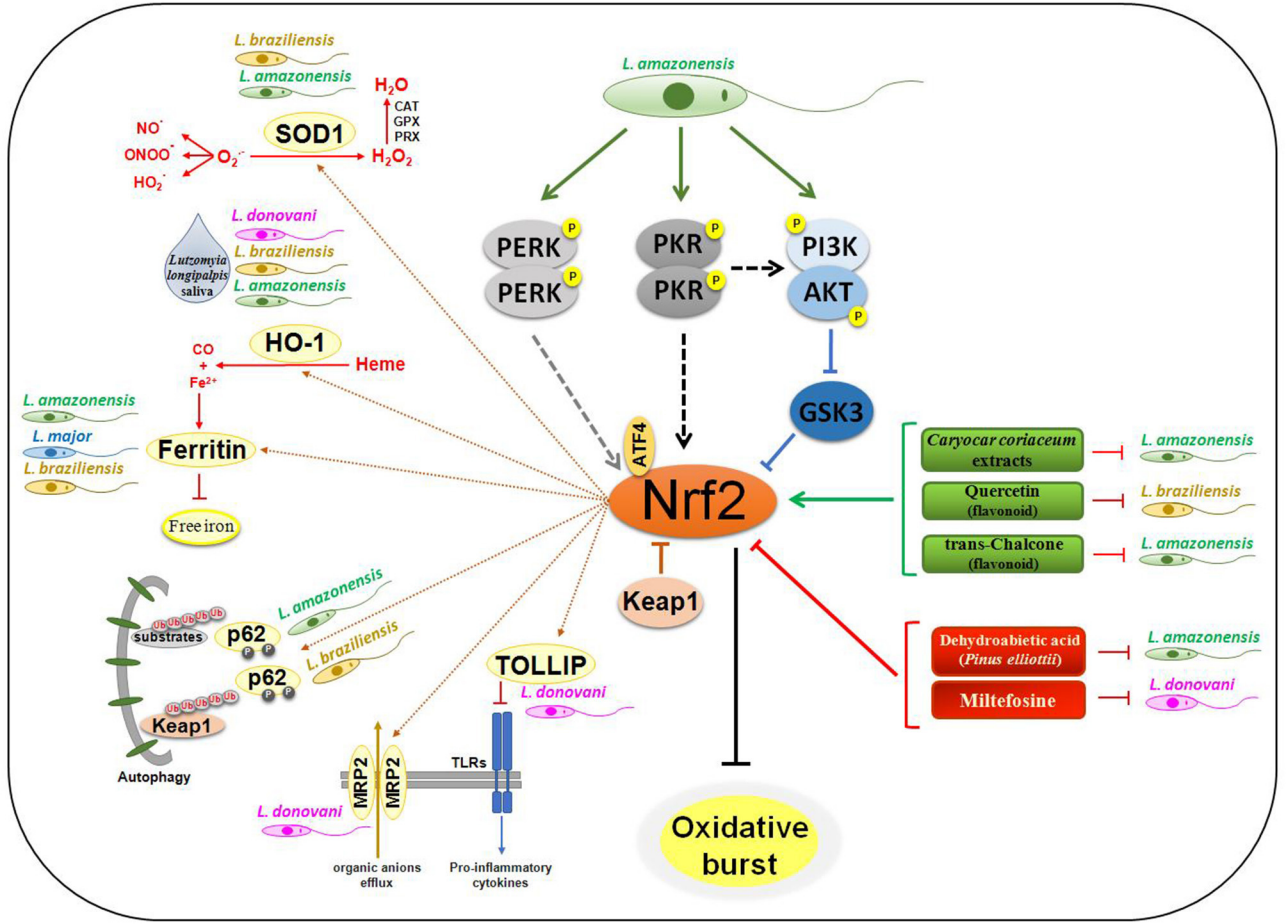
Nrf2 signaling in *Leishmania* infection. The engagement of *Leishmania* PAMPs on sensor receptors leads to activation of PERK, PKR, and PI3K/AKT in *L. amazonensis* infection, culminating in Nrf2 activation through p62-mediated autophagic degradation of Keap1, and inhibition of GSK3 activity. Flavonoid and extracts of some plants may lead to activation of Nrf2 by mechanisms not yet defined, disfavoring all infection through of Ferritin metabolism. In contrast, treatment with DHA and Miltefosine decreases Nrf2 activation and also the infection progression. The amount of oxygen free radicals and pro-oxidative molecules like Heme is drastically reduced due to the expression of SOD1 and HO-1. With the production of Fe^2+^ by HO-1, Ferritin's iron sequestering depletion the intracellular ion and interfere on replication of the parasites. Proinflammatory cytokine production by different TLRs is also reduced by increasing expression of TOLLIP, an Nrf2-dependent gene. The expression of the MRP2 transporter leads to an increase in the efflux of organic ions, making the host cell resistant to different types of products.

Nrf2 and ARE-responsive genes may comprise important targets for Leishmaniasis treatment (Figure 1). The Nrf2 signaling promotes the reduction of oxidative burst in cell host and these parasites, throughout evolution, developed mechanisms responsible for the induction of this anti oxidative pathway. Although the host cell is partly prepared to intervene in infections that break its homeostasis, *Leishmania* parasites try to subvert these signaling in the host cells and modulate important mediators for the establishment and progression of the infection.

Strong evidences data indicate that central signal transducers such as PKR, PERK, and PI3K/Akt play an important role in Nrf2-mediated anti-oxidant process in Leishmaniasis. Moreover, post-translational modifications in Nrf2 pathway components have not yet been demonstrated. No one knows whether these kinases directly phosphorylate this transcriptional factor, except for GSK3, or intermediate molecules that culminate in their activation. Continuing with the gaps, there is still to be researched which receptors in macrophages are directing for the activation of the pathway, added research to identify parasite antigens that engage in these receptors.

There are differences in the activation of Nrf2 by different *Leishmania* species, evidenced mainly between *L. amazonensis* and *L. major*. Cells infected with *L. braziliensis* have a profile very close to that found during *L. amazonensis* infection, although they belong to distinct subgenera. *L. donovani* infection also activates nuclear translocation and Nrf2 activity, decreasing oxidative stress, but there is still no evidence of which molecular partners are required to trigger this signaling. In this context, it is clear that different *Leishmania* species, besides unknown components of *Lutzomyia longipalpis* vector saliva, are directly involved in Nrf2 modulation and this has become a fundamental requirement for interaction with the host cell. What is known in concrete is that Nrf2 expression and activation occurs in initial contact with cell host increasing the amount of gene products related to an antioxidant profile and characteristics of M2 macrophages like anti-inflammatory spectrum, and that Nrf2 knockout cell or its inhibition decrease the parasite infection.

But despite the antioxidant effect on cells, continuous activation of Nrf2 can greatly decrease ROS levels, which is also essential for cellular homeostasis. One of the targets of Nrf2 is the Ferritin gene, which sequester Fe^2+^, thereby decreasing iron metabolism for parasite growth. In addition, oxidative stress may occur on parasites due to the need for the iron atom for *Leishmania*' Superoxide dismutase activity, unlike the copper-dependent enzyme in mammals.

Extracts and purified molecules of some plants have been able to ambiguously modulate the Nrf2 pathway with leishmanicidal effect or their activation by a mechanism yet to be established. But the great prospect is the use of miltefosine, the first oral drug against leishmaniasis, which may lead to suppression of Nrf2 pathway components and some target genes, changing to the pro-inflammatory macrophage profile. But, what are the intracellular targets of these products?

The tight regulation of oxidative stress and inflammation is very important to guarantee a balanced immune response without developing pathologies and is crucial for the initiation of healing. The either hypo- or hyper-activation of Nrf2 maybe contribute to the onset of chronic diseases and tumor development (Vomund et al., 2017). The dysregulation of Keap1/Nrf2 signaling promotes the dual roles of Nrf2 in cancer, being considered a tumor suppressor and a target oncogene (Menegon et al., 2016). There is rare case associations of leishmaniasis with cancer-related disorder, but nothing yet associated with Nrf2 regulation (Kopterides et al., 2007; Kumar et al., 2011). Activation of Nrf2 is also related to the recruitment and infiltration of neutrophil natural killer (NK) and monocyte cells at the site of inflammation, and this process is mediated by Interleukin (IL)-17D, a target of Nrf2 (Seelige et al., 2018). Mice deficient in the Nrf2 is characterized by a decreased number of innate leukocyte cells, implying a greater proliferation of the pathogen (Reddy et al., 2009). The question is what would be the limit of Nrf2 modulation necessary to achieve a parasite and host cell homeostasis without secondary effects that would damage both cells? This potential drawback of targeting the Nrf2 pathway is important consequences for the success of the parasite-host cell interaction.

This co-evolution, perhaps, has become a primary mechanism in the establishment of infections and, thus, draws immense attention to the understanding of the mechanisms. More research is necessary to uncover the main sensors implicated in Nrf2 activation by different *Leishmania* species. The identification of molecular partners of Nrf2, as well as the range of genes that may be involved in oxidative burst, will open perspectives to select possible therapeutic targets.

## Author Contributions

AV and UL designed the review, wrote the manuscript, and approved the final version of the manuscript.

### Conflict of Interest

The authors declare that the research was conducted in the absence of any commercial or financial relationships that could be construed as a potential conflict of interest.
